# Antifungal Activity of Propyl-Propane-Thiosulfinate (PTS) and Propyl-Propane-Thiosulfonate (PTSO) from *Allium cepa* against *Verticillium dahliae*: In Vitro and in Planta Assays

**DOI:** 10.3390/jof7090736

**Published:** 2021-09-08

**Authors:** Ana Falcón-Piñeiro, Efrén Remesal, Miguel Noguera, Juan José Ariza, Enrique Guillamón, Alberto Baños, Juan Antonio Navas-Cortes

**Affiliations:** 1 DMC Research Center, Camino de Jayena, 82, 18620 Granada, Spain; anafalcon@dmcrc.com (A.F.-P.); miguel.noguera@diesia.uhu.es (M.N.); jariza@dmcrc.com (J.J.A.); eguillamon@domca.com (E.G.); 2 Instituto de Agricultura Sostenible (IAS), Consejo Superior de Investigaciones Científicas (CSIC), 14004 Córdoba, Spain; efrenrg@kimitec.com (E.R.); j.navas@csic.es (J.A.N.-C.); 3 Centro de Investigación en Tecnología, Energía y Sostenibilidad (CITES), Universidad de Huelva, 21819 Huelva, Spain

**Keywords:** *Verticillium* wilt, onion, organosulfur compounds, olive trees, pest management

## Abstract

*Verticillium* wilt, caused by *Verticillium dahliae*, is the most devastating soil-borne fungal disease of olive trees worldwide. Currently, there is no effective measure available to control the pathogen in diseased plants in open field conditions. Searching more effective and sustainable solutions are a priority for the olive sector. The existing alternatives for disease control include the use of biological control microorganisms and compounds of natural origin from plants, such as Alliaceae. Propyl propane thiosulfinate (PTS) and propyl propane thiosulfonate (PTSO) are two organosulfur compounds derived from *Allium cepa* with a widely documented antimicrobial activity. The aim of this study was to evaluate the antifungal activity of PTS and PTSO against the defoliating and non-defoliating *V. dahliae* pathotypes. Firstly, several in vitro tests were performed (Minimum Antifungal Concentration, susceptibility studies according to the Kirby–Bauer disk-diffusion method, antifungal activity through aerial diffusion and effect on mycelial growth). The ability of both compounds to sanitize soil was evaluated using a sterile substrate inoculated with *V. dahliae*. Finally, challenges in growth chambers were carried out. PTS and PTSO generated growth inhibition zones in agar diffusion and the gas phase, and the mycelial growth of all the *V. dahliae* strains was significantly altered. The *V. dahliae* population in soil was considerably reduced after the sanitization. Finally, *in planta* assays demonstrated the ability of these compounds to reduce disease related parameters and their contribution to control the phytopathogen. In conclusion, the results showed that the PTS and PTSO from *Allium* *cepa* display in vitro and in vivo antifungal activity against *V. dahliae* and suggested that both compounds could be used as natural and environmentally friendly tools for *Verticillium* wilt management.

## 1. Introduction

Verticillium wilt caused by fungus *Verticillium dahliae* is currently considered the main and most devastating soilborne fungal disease of olive trees (*Olea europaea* L.) [1]. Although it affects more than 400 plant species, such as cotton, tomato, almond and peach, the high incidence of this disease in all Mediterranean olive-growing regions is threating olive trees and olive oil production, causing important economic losses [2]. The severity of the attacks by *V. dahliae* on olive and cotton highly depends on the virulence of the pathotype infecting the olive trees [3]. *V. dahliae* isolates can be classified into a defoliating (D) pathotype, that is highly virulent, and a nondefoliating (ND) pathotype, based on their ability to cause the defoliation of green leaves from shoots and twigs [4]. The D pathotype can be lethal to the plant and causes the extensive and early drop of infected green leaves and, eventually complete defoliation and necrosis. Besides the necrosis of inflorescences and leaf chlorosis, the ND pathotype causes olive twigs and branches without leaf shedding to dieback [5,6]. However, trees infected by the ND pathotype can show complete remission from symptoms [3].

*V. dahliae* is a strictly asexually reproducing fungus, characterized by the production of microsclerotia as resistant structures [7]. The formation of these structures is a critical factor in the survival, dissemination and epidemiology of the pathogen [8], since they can survive in soil without a host for more than 10 years [2]. Microsclerotia germinate in soil in response to root exudates, giving rise to hyphae that can penetrate the olive root and grow across the root cortex. This way, hyphae invades the xylem vessels and forms conidia that is spread by olive tree stem, giving rise to an extensive xylem colonization and functional impairment [6].

*Verticillium* wilt is a hard to control disease, since *V. dahliae* can survive in soil for years and grow confined in the host’s xylem during the parasitic phase, preventing treatments by topically application fungicides [9]. To date, the only method that has proven to be effective against this pathogen is soil disinfection with fumigants such as methyl bromide or chloropicrin. However, the potential risk involved in applying these chemicals, for both human health and the environment, has led to their banning in many countries [10,11,12]. The lack of effective fungicides, and the increasing restrictions on the use of chemical compounds in managing plant diseases, is pushing the search for new alternative methods to control *V. dahliae* [8]. An interesting and sustainable option is the use of plant-derived natural products.

In recent years, the antimicrobial activity of several organosulfur compounds such as thiosulfinates and thiosulfonates, obtained from *Allium* genus plants such as onion (*Allium cepa* L.), Welsh onion (*Allium fistulosum* L.) or garlic (*Allium sativum* L.), has been extensively studied [10,13,14,15,16]. In onion, the most common organosulfur compounds are isoalliin (S-propenyl-L-cysteine sulfoxide) and propiin (S-propyl-L-cysteine sulfoxide). When an onion is crushed or cut, propiin changes into propyl-propane thiosulfinate (PTS) due to the action of the enzyme [17]. Despite the fact PTS is more stable than other thiosulfinates such as allicin [10], it is also a labile compound that changes into dipropyl disulfide and propyl-propane thiosulfonate (PTSO) [18].

Some potential uses of PTS and PTSO in animal nutrition have been proposed because of their anti-inflammatory [19], anti-bacterial [20] and anti-coccidial properties [21]. Furthermore, their ability to modulate the gut microbiota and improve productivity in piglets and laying hens has been recently demonstrated [22,23]. In addition, both PTS and PTSO have been shown to be toxicologically safe in different studies carried out in cell models and in experimental animals [24,25,26].

While their precise mechanism of action is not yet completely understood, these onion-derived products have the following three characteristics that could explain their antimicrobial effect: (i) they react with thiol groups of the microbial metabolism altering the integrity of membranes [27]; (ii) they can react with glutathione, decreasing its intracellular levels, which entails oxidative stress and cellular apoptosis [28,29]; and (iii) they can inhibit RNA synthesis through the inhibition of RNA polymerase [16,30].

The use of organosulfur compounds from *Allium* in agriculture has been mainly focused on antifungal activity of garlic derivatives against plant pathogens such as *Botrytis cinerea*, *Penicillium expansum*, *Bipolaris sorokiniana*, *Phytophthora infestans*, *Fusarium* and *Rhizopus* spp., among others [31,32,33,34]. Nevertheless, studies that have evaluated the antifungal activity of similar derivates from onion are limited and they are mainly focused on the effectiveness of onion essential oil or aqueous extracts [35,36,37].

The aim of the present study was to evaluate the antifungal activity of volatile organosulfur compounds PTS and PTSO from *Allium cepa* against the plant pathogenic fungi *V. dahliae*, both in vitro and *in planta*, and the determination of their potential use as soil sanitizer providing a new method to control Verticillium wilt in olive trees.

## 2. Materials and Methods

### 2.1. Antifungals

PTS and PTSO (98% purity) from Allium cepa were supplied by DOMCA SAU (Granada, Spain). The pure compounds were used for in vitro assays. A blend of PTS and PTSO in proportion 1:1 (*w*/*w*) at different concentrations was used for soil sanitization tests and in planta assays.

### 2.2. Verticillium Dahliae Isolates

Three *V. dahliae* isolates were used in this study (Table 1). These isolates belong to the culture collection of the Department of Crop Protection, Institute for Sustainable Agriculture, Spanish National Research Council, Córdoba, Spain [3]. The *V. dahliae* isolates were selected as representative of the pathotypes and mycelial compatibility groups most widely distributed in southern Spain [38]. The liquid culture medium used was RPMI-1640 medium with L-glutamine, supplied by Labclinics (Barcelona, Spain). Glucose was added to a final concentration of 0.2% and the pH was adjusted to 7.0 with acid morpholine propane sulfonic (0.165 M) buffer. The solid culture medium used was Rose-Bengal agar supplied by Scharlau (Barcelona, Spain). Inoculum of *V. dahliae* isolates were prepared in saline solution.

### 2.3. Antifungal In Vitro Assays

#### 2.3.1. Agar Diffusion Test

The chosen method to study antifungal activity in solid medium was the disk-diffusion method proposed by Kirby–Bauer [39] and modified by Calvo and Asensio [40]. Sterilized cellulose discs of 6 mm of diameter (Whatman^®^ antibiotic test discs, Buckinghamshire, UK) impregnated with 20 µL of PTS or PTSO at different concentrations (2.5, 5, 10, 25 and 50 µg/µL) were placed in the center of Rose-Bengal agar plates. These plates were already inoculated with the appropriate *V. dahliae* isolate using a conidial suspension adjusted to 10^6^ CFU/mL in saline solution, so that the growth after 4 days incubation at 25 °C was confluent. The inhibition zone of fungal growth was proportional to the degree of inhibition produced. Two independent tests were carried out, and each sample was tested in duplicate. EC50 values were also calculated.

#### 2.3.2. Minimum Fungicidal Concentration (MFC)

Standard broth microdilution method was carried out according to the guidelines of the Clinical and Laboratory Standards Institute (CLSI) [41]. Decreasing concentrations of PTS and PTSO were prepared by making 1:2 dilutions from a starting concentration of 10,000 µg/mL (10,000, 5000, 2500, 1250, 625, 312.5, 156.25, 78.125, 39.06, 19.53 and 9.76 µg/mL) in RPMI-1640 medium. These dilutions were subsequently inoculated with the corresponding *V. dahliae* isolate and incubated for 24 h at 25 °C in the dark. As a negative control, same broth volume was inoculated with *V. dahliae* and incubated in the same conditions. Natamycin, a polyene macrolide with antifungal properties, was used as a positive control. All assays were performed in duplicate, and each sample was tested twice.

#### 2.3.3. Antifungal Activity of Gaseous PTS and PTSO

To study the antifungal activity of the compounds associated with their gas phase, the methodology described in Section 2.3.1 was carried out with some modifications [14,42]. *V. dahliae* colonies were suspended in saline solution to obtain a conidial suspension adjusted to 10^6^ CFU/mL that was used to inoculate the Rose-Bengal agar plates. As previously described, sterilized cellulose discs were impregnated with 20 µL of PTS or PTSO at different concentrations (2.5, 5, 10, 25 and 50 µg/µL) and placed, not in the agar, but in the center of the Petri dish lids, so that the agar plates with *V. dahliae* were placed inverted over the lid. Therefore, the tested compounds and the inoculated agar did not come into direct contact, except by diffusion through the air. After incubation at 25 °C for 4 days in the dark, the diameter of the inhibition zones was measured (mm). Assays were performed in duplicate, and each sample was tested twice. EC50 values were also calculated.

### 2.4. Effect of PTS and PTSO on Mycelial Growth

The protocol chosen to carry out this assay was based on the method proposed by Liu–Yan [43]. First, agar plates containing Rose-Bengal medium supplemented with PTS and PTSO at different concentrations (25, 50, 75 and 100 µg/mL) were prepared. Non-supplemented Rose-Bengal agar plates were used as positive control of *V. dahliae* growth. Next, 10-microliter drops of a previously titrated *V. dahliae* inoculum at 10^6^ CFU/mL were placed in the center of the agar plates, and these were incubated at 25 °C for 16 days in the dark. Mycelial growth was determined by measuring the diameter (mm) of the mycelium over time and EC50 was calculated by using data from the last day of the assay. Percentage of mycelial growth inhibition was expressed according to the following formula:

% inhibition = [(C − T)/C] × 100

where C is the diameter of the fungal colony in the control plate, and T is the diameter of the fungal colony in each treatment plate. Two independent tests were carried out and each sample was tested in duplicate.

### 2.5. Soil Sanitization

The soil used for this study was a universal substrate (Composana, Münster, Germany) that was autoclaved in a steam sterilizer (Raypa, Terrasa, Spain) at 121 °C for 20 min. Sterility of soil was confirmed by incubating on Rose-Bengal agar at 25 °C for 4 days [44]. Plastic bags were filled with 400 g of sterilized soil. Bags were inoculated by adding 10 mL of a conidial suspension of *V. dahliae* isolate V136I adjusted to 10^7^ CFU/mL, so that the final population in the inoculated soil was approximately 10^5^ CFU/g [13]. A bag without inoculum was used as negative control and another bag only with inoculated soil was used as positive control. In the remaining bags, the blend of PTS and PTSO in 1:1 proportion was applied 72 h after soil infestation at different concentrations (100, 500 and 1000 µg/mL). Bags were shaken to homogenize their content and sealed to avoid leaks and contamination. *V. dahliae* population was quantified at 1, 2, 3 and 4 days after treatment. At each time, 25 g of soil from each bag was suspended in 225 mL of buffered peptone water (Scharlau, Barcelona, Spain). A lab paddle blender (MASTICATOR, IUL, Barcelona, Spain) was used to obtain a better homogenized sample for analysis. Serial dilutions were placed in Rose-Bengal agar and incubated at 25 °C for 4 days in the dark [13]. Then, colonies were counted and the concentration *V. dahliae* in soil was expressed as Log_10_ CFU/g. Each test was carried out twice and each sample was tested in duplicate.

### 2.6. Effect of PTS and PTSO on Verticillium Wilt Suppression

*In planta* experiments were carried out in growth chambers using 7-month-old olive plantlets of cv. Picual, which grew in soil artificially infested with the highly virulent defoliating pathotype of *V. dahliae* V136I 1A, and incubated under optimal environmental conditions for Verticillium wilt development [38]. Inoculum consisted of an infested cornmeal–sand mixture (CMS; sand: cornmeal: deionized water, 9:1:2, *w*/*w*) produced as described by Jiménez-Fernández et al. [6]. The infested CMS was homogenized, dried in an incubator adjusted to 33 °C for 3 days, and thoroughly mixed with a pasteurized soil mixture (clay loam: peat, 2:1, *v*/*v*) at a rate of 1:20 (*w*/*w*) to reach an inoculum density of 10^5^ CFU/g soil of *V. dahliae* as determined by dilution-plating on chlortetracycline-amended water agar (CWA; 1 L distilled water, 20 g agar, 30 mg chlortetracycline) [6]. The *V. dahliae*-infested soil was treated with two doses of the blend of PTS/PTSO (250 and 500 µg/mL) and kept in sealed plastic bags for 2 days. Then, plants were uprooted from the potting substrate, gently shaken to retain only the rhizosphere soil and placed in 1500 mL pots filled with this *V. dahliae*-infested soil mixture. Non-inoculated plants were treated similarly and transplanted to the pasteurized soil mixture with non-infested CMS at the same rate as infested CMS. Once planted, a group of plantlets was not treated, and other groups received 100 mL of the blend of PTS/PTSO (250 and 500 µg/mL) via irrigation a week after being planted.

Both inoculated and control plants were incubated in a growth chamber adjusted to 22 ± 2 °C, 60–80% relative humidity and a 14-hour photoperiod of fluorescent light of 360 µmol m^−2^ s^−1^ for 3 months. There were 10 replicated pots (one plant per pot) for the different treatments and for the inoculated and non-inoculated plants in a completely randomized design. Disease reaction was assessed by the incidence (percentage of plants showing disease symptoms) and severity of foliar symptoms. Symptoms were assessed on individual plants on a 0 to 4 rating scale according to the percentage of affected leaves and twigs at 2- to 3-day intervals throughout the duration of the trial [6]. Upon termination of the experiment, the extent of colonization by *V. dahliae* was determined by isolation of the fungus in CWA [6]. Six-centimeter-long stem pieces sampled from the main stem were processed for the extraction of xylem microbiome at the same time. Data of pathogen isolation from the stem were used to calculate the intensity of stem vascular colonization for each individual plant, according to a stem colonization index (SCI) as described before [6].

### 2.7. Statistical Analyses

The average data standard deviations were determined with Excel software (Microsoft Corp., Redmond, WA, USA). Statistical analyses were performed using the SPSS-PC 15.0 software (SPSS, Chicago, IL, USA). Data on microbiological counts were subjected to ANOVA. Data of in soil and *in planta* assays were analyzed according to Tukey HSD test. Error probability values less than 0.05 were considered not significant.

## 3. Results

### 3.1. In Vitro Antifungal Assays

#### 3.1.1. Antifungal Activity

**Figure 1:**
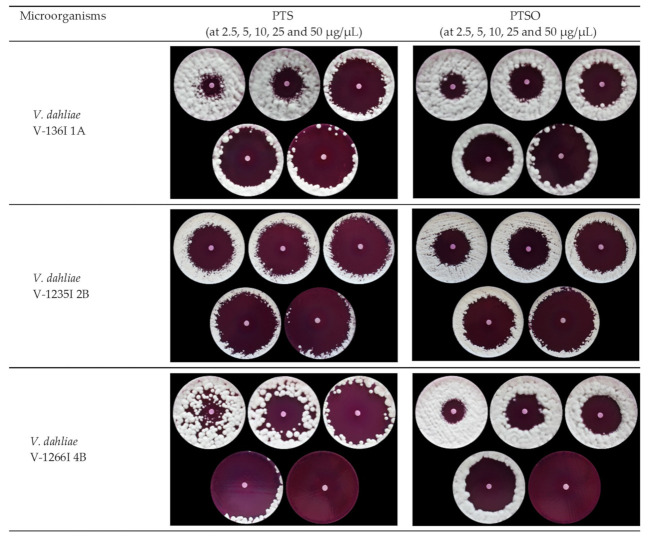
Antifungal activity of PTS and PTSO against *Verticillium dahliae* V136I 1A, V1235I 2B and V1266I 4B isolates using the disk-diffusion method. The image shows inhibition zones at doses of 2.5, 5, 10, 25 and 50 µg/µL from left to right and from top to bottom.

The antifungal activity of organosulfur compounds PTS and PTSO has been evaluated against three different isolates of *V. dahliae* (Table 1). A summary of the antifungal activity data obtained using the disk-diffusion method in agar are shown in Table 2, expressed as the average diameter ± standard deviation (in mm). All the *V. dahliae* isolates were sensitive to both compounds in a dose-dependent manner, becoming more evident from a concentration of 10 µg/µL, with inhibition zones between 50 and 87 mm. The antifungal activity of PTS and PTSO was similar in this trial, without any remarkable variations between the inhibition zones, as shown in Figure 1.

The results obtained in the agar diffusion test, which are purely qualitative, were supported by the determination of the MFC. The MFC values obtained (Table 3) confirm the potential of PTS and PTSO against *V. dahliae* isolates. Unlike in the agar diffusion test, in which no significant differences were observed between both compounds, the MFC data indicated that the antifungal susceptibility of PTSO was significantly higher than that of PTS (*p* < 0.01). This antifungal activity was especially relevant against *V. dahliae* V136I 1A, with an MFC value of 7.91 µg/mL of PTSO.

#### 3.1.2. Antifungal Activity of the Gas Phase

**Figure 2:**
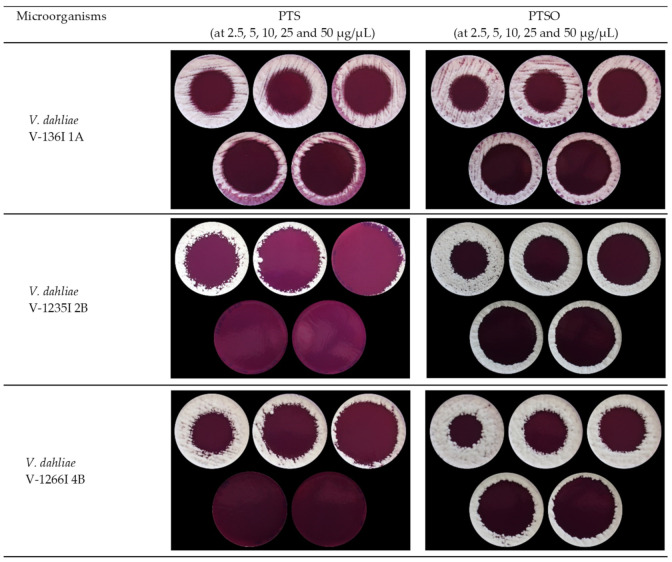
Antifungal activity of PTS and PTSO against *Verticillium dahliae* V136I 1A, V1235I 2B and V1266I 4B strains via the gas phase. The image shows inhibition zones at doses of 2.5, 5, 10, 25 and 50 µg/µL from left to right and from top to bottom.

Table 4 shows the results of the volatility-linked activity assay, expressed as the diameter of the inhibition zone ± standard deviation (in mm). PTS and PTSO inhibited growth in all the *V. dahliae* isolates tested in the present study through their gaseous phase without coming into contact with the medium and thus, with the fungus, except for its aerial diffusion. The vapor produced by both compounds reached the agar medium, inhibiting fungal growth in a circular area above the drop placed in the lid of the Petri dish (Figure 2). The absence of fungal growth suggests a predominant biocidal effect, being particularly remarkable for PTS at doses of 25 and 50 µg/µL against ND pathotypes V1235I 2B and V1266I 4B.

### 3.2. Effect of PTS and PTSO on Mycelial Growth

**Figure 3:**
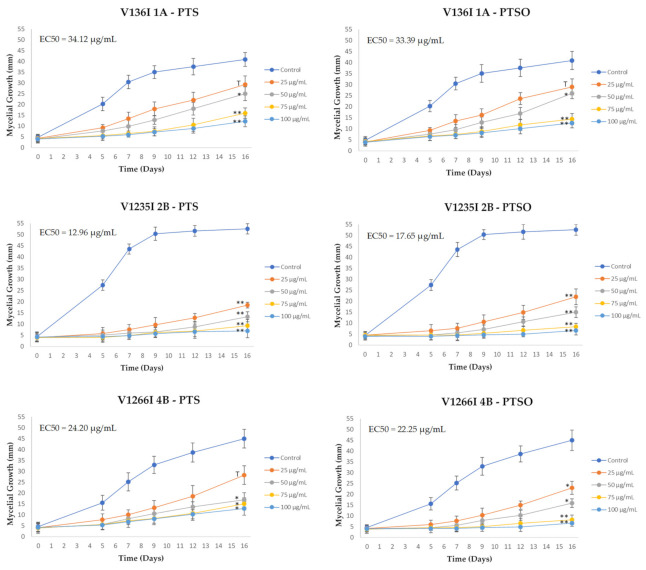
Mycelium Growth Curves of *Verticillium dahliae* isolates at different concentration of PTS and PTSO: control group; 25; 50; 75 and 100 µg/mL. Values are means with SD in bars. ^T^ *p* < 0.1; * *p* < 0.05; ** *p* < 0.01 respect to control. EC50 values have been calculated for each *V. dahliae* isolate and each organosulfur compound.

To complete the in vitro characterization of the antifungal activity studies, the fungistatic capacity was evaluated using the mycelial growth progression test in agar plates. The results obtained are presented in Figure 3. *Mycelium Growth Curves* provide additional information on the interaction between the compounds tested and the fungal growth. As in the previous assay, both PTS and PTSO showed a remarkable inhibitory effect on the *V. dahliae* mycelial growth.

These results demonstrate the correlation between the inhibition produced and the concentration of both compounds. It should be noted that the increase over time was more pronounced at the lowest concentrations tested, whereas at the concentration of 100 µg/mL, the fungal growth was practically imperceptible. The treatments of 50, 75 and 100 µg/mL of PTS or PTSO showed significant differences (*p* < 0.05), as compared to the control group. The effectiveness of PTSO at 25 µg/mL was lower against the V136I isolate. Likewise, PTS at 25 µg/mL was less effective against V136I 1A and V1266I 4B.

These results are further reflected in Table 5, which shows the inhibition of mycelium growth caused by each treatment compared to the normal growth of the control group, expressed as a percentage.

### 3.3. Application of PTS and PTSO in Soil Sanitization

The *V. dahliae* population in the untreated group (C +) evolved as expected, achieving 5 Log_10_ CFU/g on day four. In addition, the absence of growth in the negative control (sterilized soil that was not inoculated with *V. dahliae*) confirmed that sterile conditions were maintained during all of the assays.

**Figure 4:**
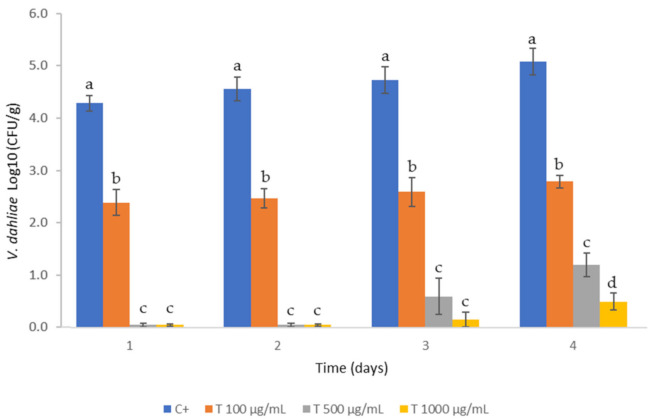
Concentration of *Verticillium dahliae* in soil after treatment with the blend of PTS and PTSO in 1:1 proportion at different concentrations (100, 500 and 1000 µg/mL) compared to a positive control group (C +) that consists of sterilized soil inoculated with *V. dahliae* but without treatment. Values are means with standard deviation in bars. For each sampling day, bars with a different letter (a–d) indicate significant differences according to Tukey HSD test at *p* < 0.05.

The different doses of the blend of PTS/PTSO achieved a significant reduction (*p* < 0.05) in the population of *V. dahliae* in soil, as shown in Figure 4. The soil treated with a dose of 100 µg/mL reduced the fungal concentration by approx. 2 Log_10_ CFU/g during the assay. The effects observed in soil treated with a dose of 500 µg/mL were more remarkable, showing an absence of growth at days one and two after treatment. Nevertheless, fungal growth was observed on days three and four, reaching 1.5 Log_10_ CFU/g. Finally, the highest inhibition occurred for the dose of 1000 µg/mL, with reductions in the counts up to 4–5 logarithmic units, as compared to the control.

### 3.4. Effect of PTS and PTSO on Verticillium Wilt Suppression

**Figure 5:**
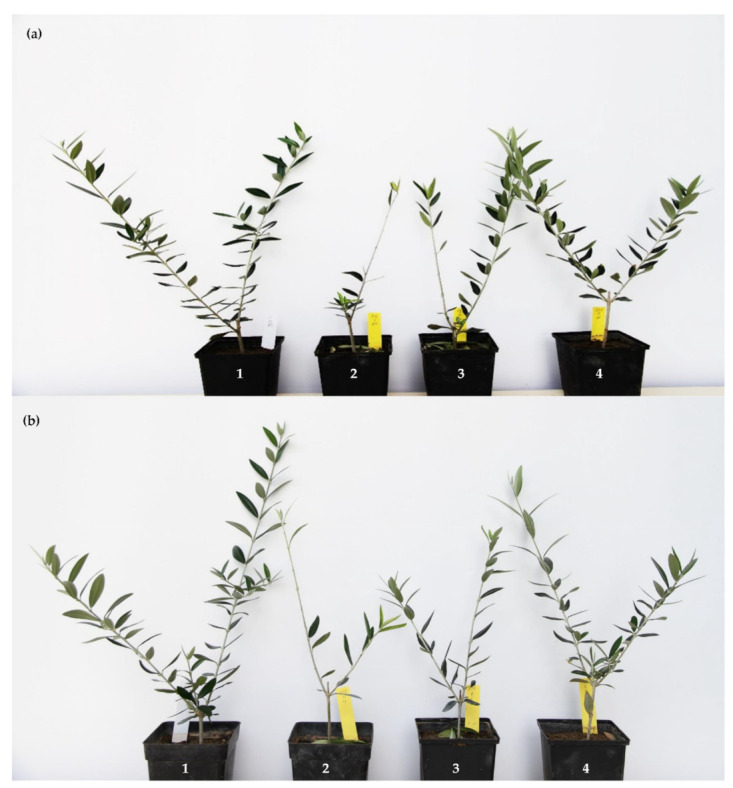
Symptoms of Verticillium wilt in olive plantlets of cv. Picual that grew for 2 months in soil infested with the defoliating pathotype V136I 1A of *Verticillium dahliae* treated with the blend of PTS and PTSO in 1:1 proportion at different concentrations: (**a**) Plants that were not further treated with AP; (**b**) Plants that were treated once with 100 mL one week later via irrigation. In both panels (**a**,**b**), the following can be observed: (1) Negative control group without fungal inoculation or treatment, (2) Positive control group that grew in soil infested with *V. dahliae* and not treated. (3) Plants that grew in *V. dahliae* infected soil treated with the blend of PTS/PTSO at 250 µg/mL and (4) Infected test group treated with the blend of PTS/PTSO at 500 µg/mL.

**Figure 6:**
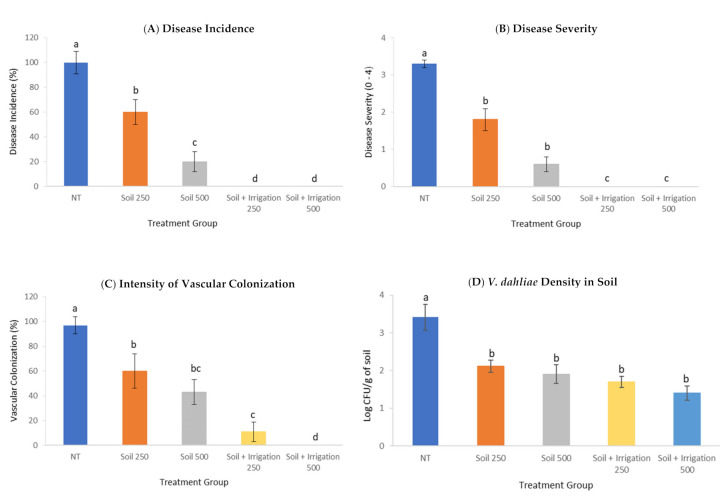
Disease reaction of olive trees of cv. Picual that grew in soil infested with the defoliating *Verticillium dahliae* (NT) pathotype treated with the blend of PTS and PTSO in 1:1 proportion at 250 and 500 µg/mL, 92 days after transplanting to the infested soil: (**A**) Disease incidence expressed as percentage, (**B**) Disease severity expressed in scale from 0 to 4, (**C**) Intensity of vascular colonization expressed as percentage, (**D**) Density of *V. dahliae* in soil expressed as Log_10_ CFU/g of soil. All panels include results from plants previously treated with the blend of PTS/PTSO or treated once with 100 mL of their respective dose one week later (Soil + Irrigation). For each panel, bars with a different letter indicate significant differences according to Tukey HSD test at *p* < 0.05.

All the soil treatments significantly reduced all the disease parameters in the study compared to that reached on the non-treated *V. dahliae* infested soil (*p* < 0.05). After 3 months of incubation under optimal environmental conditions for Verticillium wilt development, the disease incidence was 100% and the severity of Verticillium wilt symptoms reached 3.3 (on a 0–4 rating scale) in *V. dahliae*-infested but non-treated soil (Figure 5a,b and Figure 6a,b). Interestingly, the plants growing in treated infested soil showed a disease incidence of 60 and 20% (Figure 6A), a severity of symptoms of 1.9 and 0.6 (Figure 6B) and an intensity of vascular colonization of 60 and 43% (Figure 6C), on soil treated with a blend of PTS/PTSO at doses of 250 and 500 µg/mL, respectively (Figure 5a).

Moreover, no Verticillium wilt symptoms were observed on the plants growing in treated soil and further irrigated with 100 mL of the blend of PTS/PTSO one week after transplanting at any of the two doses tested (Figure 5b and Figure 6A,B). At the end of the experiment, the *V. dahliae* inoculum density was clearly reduced (Figure 6D).

Significant reductions were observed in the soil treated with 250 µg/mL of the blend of PTS/PTSO according to the Tukey test. The highest reduction was observed in the case of *V. dahliae*-infested soil treated with 500 µg/mL of the blend of PTS/PTSO and subsequently irrigated with the same product one week after planting. In this sample, the inoculum density of *V. dahliae* was reduced with differences up to two logarithmic units as compared to the infested non-treated control group (Figure 6D). Although a dose-dependent trend was observed, there were no significant differences between the four treatments in relation to the fungal concentration (Figure 6D).

## 4. Discussion

The increasing trend in the number of soilborne fungal diseases outbreaks that has been observed over the last decade, with the consequent loss in crop production and increased costs, has become a major problem for plant’s health [45]. Moreover, it has been widely reported that the proportion of soilborne pathogens and global warming are directly related, considering that the temperature determines the distribution of soil microbial communities and influences the distribution of fast-growing opportunistic fungal infections [46]. Therefore, the search for new integrated soilborne disease management strategies has become a priority.

In this context, the broad antimicrobial activity of organosulfur compounds derived from *Allium* spp. against a variety of bacteria and fungi has already been demonstrated in various in vitro studies [10,14,31,42,47]. Although the mechanism of action of organosulfur compounds is not yet deeply studied, the antimicrobial activity of these compounds could be associated with their capacity to react with the thiol groups of microbial enzymes, RNA synthesis blocking mechanisms, induction of oxidative stress and cellular apoptosis [15,29,30].

Some plant extracts from other botanical species such as *Vaccinium myrtillus* L., *Laurus nobilis* L., Eucalyptus camaldulensis Dehnh, *Mentha piperita* L., *Thymus vulgaris* L. and *Lavandula angustufolia* Mill. [48,49,50] (Bayar et al., 2018; Üstüner et al., 2018; Erdogan et al., 2016) have been shown to be able to inhibit the in vitro mycelial growth of different phytopathogenic fungi, including *V. dahliae*. Moreover, in inoculated olive plants, the essential oil of *Thymus* sp. completely inhibited mycelial growth, and reduced microsclerotia viability and Verticillium wilt disease [51].

In the present study, the antifungal efficacy of PTS and PTSO from *Allium cepa* has been evaluated against three isolates of soilborne fungus *V. dahliae*, two of them being of the non-defoliating (ND) pathotype and the third one a representative of the defoliating pathotype (D) and VCG infecting olive trees in southern Spain. Even though at the plant level, the D pathotype shows a greater virulence and lethality compared to the ND [3], our results showed no differences in the antifungal activity of PTS and PTSO between the D and ND isolates, neither in the disk-diffusion tests nor the MFC values obtained. However, the D pathotype appeared to be somewhat less affected to the gas phase of PTS and PTSO during the volatility-linked activity test, while the antifungal effect was quite more noticeable against the isolates of the ND pathotype. In line with our results, other authors have recently reported the antimicrobial activity of PTS and PTSO against bacteria and yeast through their gas phase [14].

Once the antifungal activity of PTS and PTSO in vitro tests was verified, a soil sanitization assay was performed using a preparation based on both organosulfur compounds in a 1:1 proportion. As reported by Panth et al. [45], plants belonging to *Allium* spp., such as onion, have been used in biofumigation, a soilborne pathogen management approach that consists of grinding a plant cover set up during the fallow period and incorporating the biomass obtained into the soil so that it can release active organic compounds. By using crushed onion, it has been possible to verify that the pesticidal activity of the organosulfur compounds released into the soil is comparable to that of methyl bromide. These results, described by Arnault et al. [47], not only confirmed the broad spectrum of action of onion active compounds, but also showed that they stimulate vegetative growth.

The assay carried out in sterilized soil infested with *V. dahliae* displayed very positive results in terms of disease control and the reduction in fungal concentration. In fact, PTS and PTSO reduced the microbial density of *V. dahliae* in the infested soil by up to four logarithmic units. Despite the fact that the treatment did not completely eradicate the presence of the fungus in the soil, its great effect on the fungal population was highly remarkable as a potential control measure for this disease. Deberdt et al. [13] treated soil infested with the plant pathogenic bacteria *Ralstonia solanacearum* with *Allium fistulosum* extract (Welsh onion), and found out that, although the pathogen may have survived at low levels in the soil and was even able to infect tomato roots, it was unable to fully colonize or cause observable symptoms. In the case of *Verticillium* wilt in olive trees, the progressive disease remission of symptoms has been reported, in particular when olive trees are infected by the ND pathotype. This phenomenon is called the symptomatic recovery of the disease plant, and it was first described in 1965 by Wilhelm and Taylor [52]. The recovery in conditions of moderate infection is related to the inactivation of *V. dahliae* in the xylem of the plant, with a new infection being necessary to develop the disease again [9]. Thus, reducing the severity of the infection, as well as protecting the root system of the recovered olive trees are essential for the *Verticillium* wilt integrated control approach. This could be applied to PTS and PTSO, that even though they are not able to fully eradicate the fungus in soil, the reduction produced is enough to minimize the impact of the disease in the plant.

From the results obtained *in planta* assays, it could be deduced that the organosulfur compounds from onion may improve the microbiological quality of soil; thus, further studies are needed to establish their influence on the soil microbiome and whether they can be used as a pre-plant treatment.

As previously mentioned, scientific reports on the activity of organosulfur compounds from onion against plant pathogens are limited. On the contrary, more evidence on the antifungal activity of similar compounds from garlic, such as diallyl thiosulfinate or allicin [10,42] has been found. Some authors have reported the antifungal activity of an allicin against *V. dahliae*, both in vitro and in a leaf disk bioassay [53]. Recently, Ali et al. [54] demonstrated the ability of an aqueous garlic extract containing allicin to reduce the disease severity index in eggplant seedlings against *V. dahliae*.

Even though further studies are needed, both in greenhouse and in field trials evaluating the effects of the compounds over time in a larger number of plants, in which different doses and methods of application of the compounds are assessed, the antifungal activity of PTS and PTSO against one of the most aggressive soilborne pathogens has been proven in this study, providing new knowledge on the activity of Allium organosulfur compounds and their potential use for plant disease control.

## 5. Conclusions

Both PTS and PTSO showed a remarkable ability to reduce the growth of *Verticillium dahliae*, highlighting their antifungal activity in the gas phase, which is related to the volatility of these metabolites. Moreover, these organosulfur compounds from *Allium cepa* can be an alternative to chemical soil disinfectants. Although additional studies in real field conditions are necessary to confirm their potential in integrated pest management, the results obtained after in vitro and *in planta* assays support that both compounds could be a useful and environmentally friendly tool in Verticillium wilt management.

## Figures and Tables

**Table 1:** *Verticillium dahliae* isolates used in this study, along with reference code, vegetative compatibility grouping (VCG) and pathotype.

Reference Code	VCG ^1^	Pathotype ^2^
V136I	1A	D
V1235I	2B	ND
V1266I	4B	ND

^1^ VCG = Vegetative compatibility group, ^2^ D = defoliating pathotype, ND = nondefoliating pathotype.

**Table 2:** Antifungal activity of PTS and PTSO against different *Verticillium dahliae* isolates (V136I 1A, V1235I 2B and V1266I 4B) using the disk-diffusion method, expressed as the average diameter ± standard deviation of inhibition zone (mm), and EC50 values.

Compound	Concentration (µg/µL)	V136I 1A	V1235I 2B	V1266I 4B
PTS	2.5	32.0 ± 3.16	47.3 ± 2.86	37.8 ± 3.49
	5	44.0 ± 3.16	56.0 ± 1.87	48.8 ± 2.38
	10	61.3 ± 2.38	64.5 ± 2.69	68.3 ± 3.34
	25	69.0 ± 1.41	72.3 ± 2.59	80.8 ± 1.92
	50	75.3 ± 1.92	80.5 ± 2.18	86.0 ± 3.16
	EC50 (µg/µL)	7.7 ± 0.03	10.0 ± 0.56	8.3 ± 0.17
PTSO	2.5	28.3 ± 2.38	40.0 ± 3.16	28.8 ± 3.34
	5	38.8 ± 2.38	48.8 ± 0.83	42.3 ± 2.59
	10	50.8 ± 2.38	56.5 ± 1.12	65.0 ± 3.24
	25	58.5 ± 1.12	65.0 ± 3.39	76.3 ± 1.64
	50	71.5 ± 3.04	75.8 ± 3.03	87.3 ± 0.83
	EC50 (µg/µL)	10.8 ± 0.35	11.6 ± 0.42	8.8 ± 0.24

**Table 3:** Minimum Fungicidal Concentration (MFC) against different isolates of *Verticillium dahliae*.

MFC (µg/mL)		
Isolates	PTS	PTSO
*V. dahliae* V136I 1A	78.13	19.53
*V. dahliae* V1235I 2B	78.13	39.06
*V. dahliae* V1266I 4B	78.13	39.06

**Table 4:** In vitro antifungal activity of PTS and PTSO against different isolates of *Verticillium dahliae* (V136I 1A, V1235I 2B and V1266I 4B) via the gas phase, expressed as the average diameter ± standard deviation of inhibition zone (mm), and EC50 values.

Compound	Concentration (µg/µL)	V136I 1A	V1235I 2B	V1266I 4B
PTS	2.5	48.5 ± 1.12	55.0 ± 1.41	45.0 ± 1.41
	5	53.8 ± 3.49	71.5 ± 2.06	58.0 ± 2.24
	10	63.8 ± 3.49	79.3 ± 1.30	72.0 ± 1.41
	25	69.0 ± 2.12	87.0 ± 1.58	84.3 ± 1.92
	50	75.3 ± 2.86	92.5 ± 2.06	97.3 ± 1.92
	EC50 (µg/µL)	9.9 ± 0.29	7.0 ± 0.15	10.3 ± 0.09
PTSO	2.5	38.5 ± 1.12	40.5 ± 2.06	39.0 ± 1.58
	5	46.0 ± 1.41	52.3 ± 1.48	48.5 ± 1.12
	10	54.5 ± 2.96	62.3 ± 1.92	54.3 ± 1.30
	25	62.3 ± 1.79	69.0 ± 1.58	66.0 ± 1.87
	50	66.3 ± 2.28	73.0 ± 2.24	71.5 ± 1.66
	EC50 (µg/µL)	8.8 ± 0.22	7.2 ± 0.08	9.9 ± 0.11

**Table 5:** Percentage of mycelial growth inhibition of *Verticillium dahliae* isolates at different concentration of PTS and PTSO: control group; 25; 50; 75 and 100 µg/mL.

Compound	Concentration (µg/µL)	V136I 1A	V1235I 2B	V1266I 4B
PTS	25	28.39	64.87	37.16
	50	38.85	74.74	62.40
	75	60.86	82.26	66.43
	100	70.09	86.71	71.27
PTSO	25	29.22	58.13	48.93
	50	36.32	71.48	64.62
	75	64.84	84.09	81.77
	100	69.17	87.38	85.17
